# Supplementary material for: The Relationship between Wellbeing, Self-Determination, and Resettlement Stress for Asylum-Seeking Mothers Attending an Ecosocial Community-Based Intervention: A Mixed-Methods Study
Source: Int J Environ Res Public Health. 2023 Nov 17;20(22):7076. doi: 10.3390/ijerph20227076 (PMC10671536; doi:10.3390/ijerph20227076)
Supplement: Supplementary file 1 [file ijerph-20-07076-s001.zip › ijerph-2601923-supplementary.pdf]

Table S1: Preliminary analyses

Hierarchical regression analysis on predictors for subjective wellbeing

Predictor	B	SE B	ß	t	p	VIF	ΔR^2	R^2
**Model 1**								-.080
Constant	4.316	2.131		2.025	0.053			
Age	-0.007	0.035	-0.047	-0.211	0.835	1.654		
Education	-0.119	0.500	-0.050	-0.238	0.814	1.474		
Civil status								
6 - 1	0.141	1.900	0.020	0.074	0.942	2.456		
4 - 1	-0.362	1.828	-0.052	-0.198	0.845	2.273		
3 - 1	1.774	2.399	0.253	0.740	0.466	3.914		
2 - 1	0.133	1.328	0.036	0.100	0.921	4.395		
No. of children	0.000	0.218	0.000	-0.002	0.999	1.343		
Length of stay	-0.103	0.049	-0.783	-2.101	0.045	4.634		
Program attendance	0.434	0.199	0.657	2.183	0.038	3.022		
**Model 2**							0.096	0.016
Constant	1.620	2.477		0.654	0.519			
Age	0.002	0.034	0.012	0.055	0.957	1.689		
Education	-0.130	0.477	-0.054	-0.271	0.788	1.474		
Civil status								
6 - 1	-0.344	1.831	-0.049	-0.188	0.853	2.504		
4 - 1	-0.265	1.745	-0.038	-0.152	0.881	2.275		
3 - 1	1.524	2.293	0.218	0.665	0.512	3.927		
2 - 1	0.478	1.280	0.131	0.374	0.712	4.485		
No. of children	0.049	0.210	0.045	0.235	0.816	1.364		
Length of stay	-0.081	0.048	-0.616	-1.679	0.105	4.917		
Program attendance	0.371	0.192	0.562	1.928	0.065	3.113		
Self-Efficacy	0.644	0.337	0.356	1.909	0.067	1.271		
**Model 3**							0.334	0.350
Constant	-1.327	2.159		-0.614	0.544	-1.327		
Age	0.012	0.028	0.075	0.425	0.674	0.012		
Education	-0.086	0.388	-0.036	-0.220	0.827	-0.086		
Civil status								
6 - 1	0.564	1.508	0.081	0.374	0.712	2.569		
4 - 1	0.146	1.423	0.021	0.103	0.919	2.288		
3 - 1	4.092	1.984	0.584	2.062	0.050	4.447		
2 - 1	1.256	1.061	0.344	1.184	0.247	4.660		
No. of children	0.019	0.171	0.018	0.112	0.912	1.367		
Length of stay	-0.140	0.042	-1.056	-3.301	0.003	5.668		
Program attendance	0.602	0.168	0.913	3.585	0.001	3.588		
Self-Efficacy	0.119	0.307	0.066	0.387	0.702	1.596		
Need satisfaction	0.574	0.152	0.621	3.784	<0.001	1.488		

$N = 38$

Hierarchical regression analysis on predictors for adaptive stress

Predictor	B	SE B	ß	t	p	VIF	ΔR²	R²
**Model 1**								-.136
Constant	1.840	1.062		1.733	0.095			
Age	-0.016	0.018	-0.212	-0.889	0.382	1.753		
Education	0.081	0.256	0.071	0.316	0.754	1.545		
Civil status								
6 - 1	1.101	0.948	0.329	1.161	0.256	2.473		
4 - 1	0.422	0.909	0.126	0.464	0.646	2.274		
3 - 1	0.533	1.196	0.159	0.445	0.660	3.934		
2 - 1	0.480	0.660	0.274	0.727	0.474	4.382		
No. of children	0.060	0.109	0.115	0.552	0.586	1.343		
Length of stay	0.019	0.025	0.299	0.750	0.460	4.887		
Program attendance	-0.134	0.101	-0.423	-1.329	0.196	3.117		
**Model 2**							0.007	-0.129
Constant	2.650	1.299		2.040	0.052			
Age	-0.020	0.019	-0.259	-1.073	0.294	1.813		
Education	0.094	0.256	0.082	0.368	0.716	1.548		
Civil status								
6 - 1	1.258	0.957	0.376	1.315	0.200	2.532		
4 - 1	0.389	0.907	0.116	0.428	0.672	2.276		
3 - 1	0.592	1.194	0.177	0.496	0.624	3.942		
2 - 1	0.376	0.665	0.215	0.565	0.577	4.476		
No. of children	0.046	0.109	0.089	0.423	0.676	1.362		
Length of stay	0.013	0.026	0.209	0.516	0.611	5.101		
Program attendance	-0.119	0.102	-0.375	-1.170	0.253	3.179		
Self-Efficacy	-0.191	0.177	-0.219	-1.076	0.292	1.279		
**Model 3**							0.394	0.265
Constant	4.245	1.129		3.760	<.001			
Age	-0.027	0.015	-0.354	-1.800	0.085	1.842		
Education	0.094	0.206	0.082	0.453	0.654	1.548		
Civil status								
6 - 1	0.822	0.780	0.245	1.053	0.303	2.588		
4 - 1	0.163	0.734	0.049	0.222	0.826	2.291		
3 - 1	-0.772	1.028	-0.231	-0.751	0.460	4.491		
2 - 1	-0.038	0.548	-0.021	-0.069	0.946	4.660		
No. of children	0.063	0.088	0.122	0.718	0.479	1.365		
Length of stay	0.045	0.022	0.716	2.025	0.054	5.949		
Program attendance	-0.246	0.089	-0.774	-2.775	0.011	3.706		
Self-Efficacy	0.073	0.159	0.084	0.462	0.648	1.582		
Need satisfaction	-0.299	0.079	-0.674	-3.796	<0.001	1.501		

$N = 38$
